# Text Messages Sent to Household Tuberculosis Contacts in Kampala, Uganda: Process Evaluation

**DOI:** 10.2196/10239

**Published:** 2018-11-20

**Authors:** Amanda J Meyer, Diana Babirye, Mari Armstrong-Hough, David Mark, Irene Ayakaka, Achilles Katamba, Jessica E Haberer, J Lucian Davis

**Affiliations:** 1 Department of Epidemiology of Microbial Diseases Yale School of Public Health Yale University New Haven, CT United States; 2 Uganda Tuberculosis Implementation Research Consortium Makerere University Kampala Uganda; 3 Clinical Epidemiology Unit Makerere University Kampala Uganda; 4 Harvard Medical School and Massachusetts General Hospital Boston, MA United States; 5 Pulmonary, Critical Care, and Sleep Medicine Section Yale School of Medicine Yale University New Haven, CT United States

**Keywords:** Africa, fidelity, implementation, intervention, short message service, tuberculosis

## Abstract

**Background:**

Previous studies have reported the inconsistent effectiveness of text messaging (short message service, SMS) for improving health outcomes, but few have examined to what degree the quality, or “fidelity,” of implementation may explain study results.

**Objective:**

The aim of this study was to determine the fidelity of a one-time text messaging (SMS) intervention to promote the uptake of tuberculosis evaluation services among household contacts of index patients with tuberculosis.

**Methods:**

From February to June 2017, we nested a process evaluation of text message (SMS) delivery within the intervention arm of a randomized controlled trial of tuberculosis contact investigation in Kampala, Uganda. Because mobile service providers in Uganda do not provide delivery confirmations, we asked household tuberculosis contacts to confirm the receipt of a one-time tuberculosis-related text message (SMS) by sending a text message (SMS) reply through a toll-free “short code.” Two weeks later, a research officer followed up by telephone to confirm the receipt of the one-time text message (SMS) and administer a survey. We considered participants lost to follow-up after 3 unsuccessful call attempts on 3 separate days over a 1-week period.

**Results:**

Of 206 consecutive household contacts, 119 had a text message (SMS) initiated from the server. While 33% (39/119) were children aged 5-14 years, including 20% (24/119) girls and 13% (15/119) boys, 18 % (21/119) were adolescents or young adults, including 12% (14/119) young women and 6% (7/119) young men. 50% (59/119) were adults, including 26% (31/119) women and 24% (28/119) men. Of 107 (90%) participants for whom we could ascertain text message (SMS) receipt status, 67% (72/107) confirmed text message (SMS) receipt, including 22% (24/107) by reply text message (SMS) and 45% (48/107) during the follow-up telephone survey. No significant clinical or demographic differences were observed between those who did and did not report receiving the text message (SMS). Furthermore, 52% (56/107) reported ever reading the SMS. The cumulative likelihood of a text message (SMS) reaching its target and being read and retained by a participant was 19%.

**Conclusions:**

The fidelity of a one-time text message (SMS) intervention to increase the uptake of household tuberculosis contact investigation and linkage to care was extremely low, a fact only discoverable through detailed process evaluation. This study suggests the need for systematic process monitoring and reporting of implementation fidelity in both research studies and programmatic interventions using mobile communications to improve health.

## Introduction

Mobile phone ownership in sub-Saharan Africa has increased exponentially over the last decade, becoming widespread even among those in the lowest strata of household income [1,2]. The concurrent emergence of low-cost, easy-to-use mobile health (mHealth) apps like telephone-based short message service (SMS) text messaging and chat apps like WhatsApp have facilitated a variety of new interventions to enhance communication between patients and health care providers [3-5]. While communicating health information through mobile phones seems to be acceptable [6-8], mHealth studies have reported varying levels of success at improving patient well-being and clinical outcomes [9-16]. We hypothesize that this variability may arise from a failure to plan for, collect, or report key process measures that would help differentiate between intervention failure and implementation failure, a distinction critical to understanding the feasibility and effectiveness of mHealth interventions [17]. There is a great need, both in research studies and routine practice, for carefully performed evaluations of the *fidelity* of mHealth technologies to better understand the processes and contexts that mediate their effects on patient-centered outcomes.

Process evaluations seek to understand the degree to which interventions are delivered as actually intended—also referred to as the “fidelity” of the intervention—to explain why they do or do not work and how they can be adapted to fit the local context [18,19]. Process evaluation studies may (1) measure the dose, frequency, and quality of interventions as actually delivered; (2) assess participants’ responses to interventions; and (3) characterize the mechanisms through which interventions work to improve outcomes [20]. For a health-communication intervention, a multistage process evaluation can help determine (1) if the messages reach their intended recipient; (2) if they are delivered in an accessible and timely manner; (3) if recipients open and read the messages; (4) if they respond to the messages; and (5) if the messages achieve their desired effects on targeted health behaviors. We carried out such a process evaluation of the delivery of SMS text messages in Uganda within a randomized, controlled trial of an SMS text messaging intervention designed to communicate results of household-based tuberculosis evaluation and promote the completion of follow-up procedures.

## Methods

### Study Population and Setting

**Figure 1:**
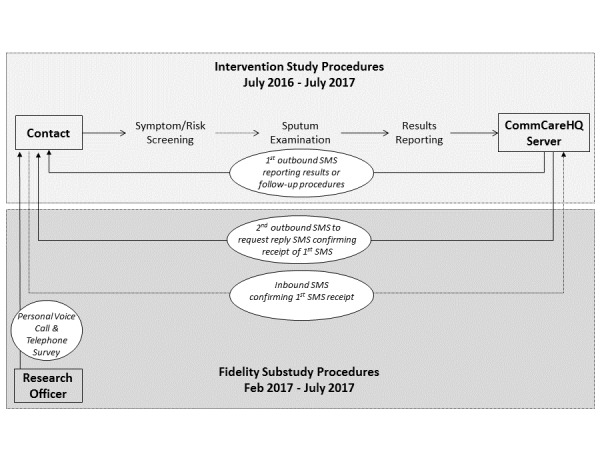
Schema for the short message service (SMS) fidelity study.

From February to July 2017, we conducted a cross-sectional study to assess rates of the receipt of SMS, time to delivery of SMS, and retention of SMS text message content among household tuberculosis contacts (Figure 1) to evaluate the fidelity of a one-time SMS text messaging intervention. We nested this study within a randomized trial (called the “parent study”) of an automated SMS text messaging intervention. The goal of the intervention was to promote the uptake of tuberculosis evaluation services among household contacts of index patients with tuberculosis at 7 public, primary care clinics in Kampala, Uganda, a setting with a high burden of undiagnosed tuberculosis [21]. In prior work in these communities, we have found that only 20% of eligible household contacts follow up in clinics to complete the tuberculosis evaluation; contacts report tuberculosis-related stigma, distrust of clinic staff, and concerns about the time and money needed to visit clinics as the main reasons for low rates of follow-up [22,23]. Although smartphones are uncommon in Uganda, we also found that almost all household members had access to mobile phones, and SMS text messages were deemed a highly acceptable way of transmitting personal health information [24]. Thus, we designed an intervention consisting of home sputum collection by community health workers (CHWs) and reporting of results by SMS text message to address these barriers to evaluation [25]. In the current process evaluation, we enrolled consecutive household contacts that were randomized into the intervention arm of the parent study.

### Procedures for Screening Household Contacts for Tuberculosis in the Parent Study

According to the parent study protocol (Pan-African Clinical Trials Registration #201509000877140), participants were eligible if they were (1) household contacts of an index patient with tuberculosis; (2) able to provide informed consent; (3) not receiving tuberculosis treatment at the baseline; (4) able to access a mobile phone; (5) willing to receive SMS text messages containing personal health information; and (6) able to speak English or Luganda, the 2 most common languages in Kampala. After obtaining written informed consent from adults and parents or guardians of minors, as well as assent from minors aged 8-17 years, CHWs screened household contacts for symptoms and other indications for the evaluation for active tuberculosis. CHWs recorded clinical and demographic information using electronic tablets equipped with a customized survey app (CommCare, Dimagi, Boston, MA, USA) wirelessly linked to a remote, cloud-based server (CommCareHQ, Dimagi). Afterwards, CHWs helped contacts register each mobile phone number to be used for SMS text messaging (described below) on the remote server by entering a registration code sent to the handset. Based on the screening results, CHWs carried out additional procedures to evaluate contacts for tuberculosis and HIV [26].

### Procedures for Short Message Service Text Messaging Within the Parent Study

CHWs recorded the results of sputum examination in the survey app, where we deployed an automated algorithm to process the relevant clinical data and assign each contact to 1-4, mutually exclusive clinical categories based on the required follow-up actions (Multimedia Appendix 1). Staff verified the logic underlying category assignments through systematic quality assurance testing of all input choices and outcomes using simulated data. We then programmed the mobile survey app to deliver a category-specific SMS text message of ≤145 characters (Multimedia Appendix 1), through integrated text messaging software (CommCare Messaging, Dimagi). The content of the messages was developed through household focus groups and interviews, as described previously [24]. Each message addressed participants by their name in English or Luganda according to their preference and provided the results of tuberculosis screening and testing with instructions for follow-up. All participants within the intervention arm of the parent study were eligible to receive SMS. All messages sent or received as part of the study were delivered free of charge to study participants.

### Process Evaluation

For the process evaluation, we scheduled a second SMS text message to be sent to all household tuberculosis contacts eligible to receive SMS text message 5 minutes after the first SMS. The second SMS text message requested that participants confirm the receipt of the original SMS text message by sending an SMS text message reply through a toll-free, 4-digit “short code.” Two weeks later, a research officer (DB) telephoned all participants (or parent or guardian for children aged <15 years), including those who had not responded to the SMS. After obtaining verbal consent, the research officer administered a short survey to confirm the SMS text message receipt and assess whether they recalled the information contained in the SMS. In addition, the research officer qualitatively recorded any unprompted comments made by participants about their overall experiences and interactions with SMS. We considered a participant a nonrespondent if he or she did not respond to the SMS text message within 2 weeks. We considered participants lost to follow-up after 3 unsuccessful call attempts on 3 separate days over a 1-week period.

### Process Measurements

To measure the fidelity of the SMS text message intervention, we specified the following 4 steps in the delivery of an SMS, each measured either by direct observation or surveying participants: (1) SMS text message sent from the CommCare Messaging server; (2) SMS text message received at the mobile handset; (3) SMS text message read by the recipient; and (4) SMS text message understood and retained by the recipient. We measured the completion of Step 1 directly from server logs; of Step 2, either by receipt of a reply SMS text message at the text messaging server or by surveying nonresponders by telephone; and of Steps 3 and 4, by surveying all participants by telephone (see Multimedia Appendices 2 and 3 for the survey content). A study epidemiologist (AJM) and a study social scientist (MAH) independently compared all responses to the retention questions about the SMS text message content.  If a participant could report back any part of the message communicated within the SMS, he or she was considered to have retained the content. Furthermore, we measured the time to delivery of the SMS text message and time to reply among respondents. As a proxy measure for the time to complete Step 2, CHWs recorded the time to delivery of the SMS text message at the time of participant registration.

### Statistical Analysis

We performed univariate analyses of participants’ characteristics. We described the number of participants for whom SMS text messages successfully reached a given step in the delivery cascade as a proportion of the number of participants for whom SMS text messages successfully reached the previous step. We performed bivariate and multivariate analyses between participants’ characteristics and completion of 3 process measures of interest—the proportion of participants who reported the SMS text message as received (Step 2), the proportion of participants from whom a reply SMS text message was sent, and the proportion of participants reporting that the SMS text message was understood and retained (Step 4). We assessed the significance of these comparisons using chi-square tests for categorical variables, and the Wilcoxon rank-sum test for continuous variables. We fit multivariate, mixed-effects logistic regression models for the same outcomes. In addition, we included all variables clinically significant in stepwise backward logistic regression models at *P*<.2 and, subsequently, adjusted for household-level clustering in a mixed-effects model. Although the sample size was based on convenience, we constructed 95% CIs to assess the precision of all study measurements. Finally, we estimated the cumulative reach of the SMS text messages (ie, of an SMS text message being sent, received, read, and retained) as the product of the proportions of participants, confirmed as reaching each subsequent step in the cascade, excluding those with unknown responses from the point of missingness. We performed all analyses using STATA version 14.2 (Stata Corporation).

### Protection of Human Subjects

This study protocol was approved by the School of Medicine Research Ethics Committee at Makerere University, the Uganda National Council for Science and Technology, and the Yale University Human Investigation Committee.

## Results

### Step 1: Short Message Service Text Message Sent

**Figure 2:**
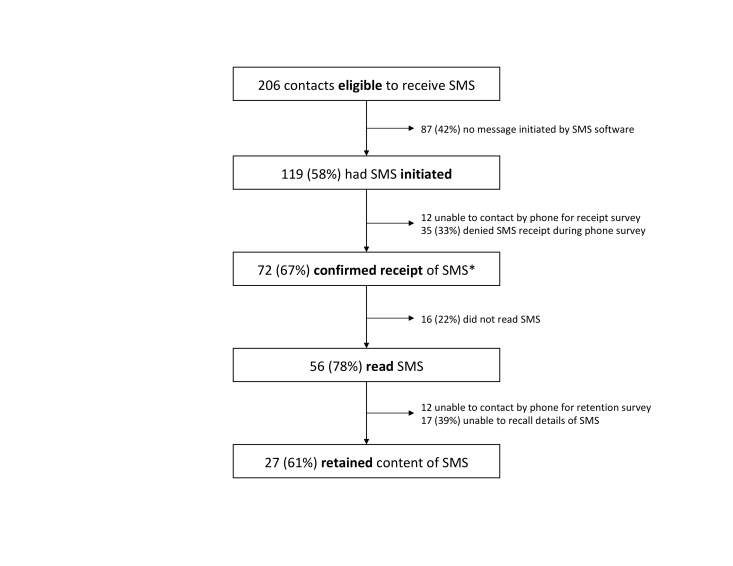
Flow diagram showing process measures for short message service (SMS) delivery. *24 participants confirmed receipt by reply SMS and an additional 48 confirmed receipt during the telephone survey.

Of 206 consecutive household contacts randomized into the intervention arm of the parent study, 58% (119/206) were sent an automated SMS text message containing tuberculosis-related information from the CommCare platform (Figure 2). A total of 42% (87/206) of participants did not have a message sent by the server, for 3 different reasons. First, 38% (79/206) of participants in the “tuberculosis visit pending” category did not have an SMS text message initiated because the delivery was erroneously posttimed owing to a programming error introduced while initiating this process evaluation substudy; this affected 25% (51/206) children aged <5 years, 7% (15/206) persons living with HIV, and 6% (13/206) individuals requiring a follow-up visit because of inadequate sputum collection. Second, 2% (4/206) of eligible participants did not have a message initiated because of missing data. Third, 2% (4/206) of participants did not have a message initiated because of server-related errors.

### Study Population of the Process Evaluation

In this study, 33% (39/119) participants who were sent an SMS text message were children aged 5-14 years, including 20% (24/119) girls and 13% (15/119) boys, whereas 18% (21/119) were adolescents or young adults, 12% (14/119) young women and 6% (7/119) young men. In addition, 50% (59/119) were adults, 26% (31/119) women and 24% (28/119) men. The median age among adult participants was 27 years, interquartile range (IQR): 21-37. Of note, 6% (7/119) of the participants who had an SMS text message sent reported tuberculosis symptoms at the time of the interview. While 55% (66/119) preferred to receive SMS text message in English, the remainder preferred Luganda. While 60% (71/119) of the participants personally owned a mobile phone registered for the study, the remainder shared the phone with close relatives (32/119, 27%), other household members (13/119, 11%), or close friends (3/119, 3%).

We reached and interviewed 80% (95/119) of participants for follow-up telephone surveys; an additional 10% (12/119) of participants responded by SMS text message but could not be reached for the telephone survey. Furthermore, 10% (12/119) of participants did not respond by SMS text message and could not be reached by telephone, leaving 107 participants with information about SMS text message receipt.

### Step 2: Short Message Service Text Message Received

Of 107 participants who had available information on SMS text message receipt, 67% (72/107) confirmed receiving an SMS, including 22% (24/107) by reply SMS text message and 45% (48/107) during the follow-up phone survey (Figure 2). No significant differences were noted in demographic characteristics between those who reported receiving the SMS text message and those who did not (Table 1). However, household contacts without tuberculosis symptoms reported receiving the SMS text message more frequently than those with tuberculosis symptoms when adjusting for household effects (69% vs 43%, cluster- adjusted odds ratio, OR, 2.9, 95% CI 0.61-13.9, *P*=.16), possibly because of chance.

**Table 1:** Demographic characteristics of participants, stratified by whether the short message service was received by the participant.

Characteristic^a^			SMS^b^ text message received by the participant (n=72)		SMS text message not received by the participant (n=35)		*P* value^c^
Female, n (%)			41 (57)		20 (57)		.98
**Age, n (%)**						.57	
	5-14 years	24 (33)		12 (34)			
	15-21 years	14 (19)		4 (11)			
	>21 years	34 (47)		19 (54)			
Tuberculosis symptoms present, n (%)			3 (4)		4 (11)		.15
**Language of SMS, n (%)**						.59	
	English	39 (54)		17 (49)			
	Luganda	33 (46)		18 (51)			
Phone owner, n (%)			43 (60)		19 (54)		.59

^a^For 107 participants with definitive SMS text message receipt status; 87 participants were excluded because SMS text message was not sent and 12 were unreachable for phone survey.

^b^SMS: short message service.

^c^Chi-square tests of significance used unless otherwise noted.

**Table 2:** Quotations from participants on their experiences with short message service text messages.

Emergent themes	Quotations^a^ from participants
Difficulty reading	“[I] did not read it because [I] was busy but [I] will ask my daughter to read it for [me].” “I could not have read it, I am old. However, I asked the CHW^b^ and they told me I didn’t have TB^c^.” “I am not good at reading. It asked me about TB symptoms.”
Phone sharing	“[I] only received the [registration] code but [I] am not the owner of the phone [my] wife is.”
Interactions with phones	“I didn’t receive it because my phone has been having some problems.” “I did not pay much attention to the SMS^d^ messages that come in.” “I don’t usually check my SMS unless someone tells me they will be sending me a message.” “Maybe the message came but I just didn’t notice.”

^a^Quotes documented by a research officer during the phone survey.

^b^CHW: community health worker.

^c^TB: tuberculosis.

^d^SMS: short message service.

### Short Message Service Text Message Reply Sent

No significant differences were observed in response to the SMS text message confirmation request by age, gender, or tuberculosis symptoms. However, those who preferred English as the language for SMS text messages were more likely to confirm receipt through an SMS text message than those who preferred Luganda (27% vs 11%, cluster-adjusted OR 3.8, 95% CI 1.03-14, *P*=.045). Similarly, those who personally owned a mobile phone were substantially more likely to respond with an SMS text message confirming the message receipt than those who shared a mobile phone with another individual (28% vs 8%, cluster-adjusted OR 6.7, 95% CI 1.3-34.9, *P*=.03). In a household-adjusted multivariate model (see Multimedia Appendix 4) including age, SMS text message language preference, and phone ownership, only phone ownership was significantly associated with increased odds of sending an SMS text message reply (adjusted OR 13.2, 95% CI 1.67-104, *P*=.01).

### Time to Delivery of the Short Message Service Text Message

Of 119 participants who had a tuberculosis-related SMS text message sent from the CommCare server, all (n=119, 100%) were sent a registration SMS text message during the initial interview with CHWs. Most registration SMS text messages (72/119, 61%) took <5 minutes to arrive at the handset. An additional 10% (12/119) took from 5-10 minutes to arrive, while 20% (24/119) took >10 minutes. Finally, 9% (11/119) of registration messages were reported as never having arrived at the handset. For 24 individuals who sent a reply SMS text message in response to the first tuberculosis-related SMS, the median time between the SMS text message being sent and a participant sending a reply message was 35 (IQR 4-139) minutes, with all but one responding within 24 hours.

### Step 3: Short Message Service Text Message Read

Of 107 participants for whom the message receipt could be determined, 52% (56/107) reported ever reading the tuberculosis-related SMS. No significant differences were observed by age, gender, tuberculosis symptoms, phone ownership, or SMS text message language preference between those who read and received and those who did not read or receive the SMS.

### Step 4: Short Message Service Text Message Retained

Overall, 61% (27/44) of individuals who reported reading the SMS text message and who participated in the retention survey were able to accurately report the details of the message when prompted. No demographic or clinical characteristics were significantly associated with SMS text message retention. However, during the phone interviews, several individuals reported having difficulty or an aversion to reading as a reason for not having read the SMS text message (Table 2). In addition, several participants described how sharing a phone prevented the intended recipient from receiving the SMS text message. Finally, having a poorly functioning phone or lacking comfort with retrieving SMS text messages influenced both SMS text message receipt and the likelihood of a participant reading an SMS text message.

### Cumulative Reach and Retention of the Short Message Service Text Message

The cumulative likelihood of an SMS text message reaching its target and being read and retained by participants was 19%. Among those for whom the text messaging server successfully initiated an SMS text message, the cumulative likelihood of receipt and retention was 32%.

## Discussion

The potential for mobile phones to improve access to evaluation and treatment for tuberculosis by enhancing communication between at-risk individuals and health care workers has generated great enthusiasm for mHealth technologies, especially SMS text messages [27,28]. However, data about how these interventions actually work—or do not work—in routine practice are limited [29]. In this study, we applied the powerful approach of measuring the implementation fidelity of SMS text messages through cascade analysis, achieved through prospective cross-sectional surveys and other novel measures of SMS text message delivery, response, and mechanism. This study shows that multiple, frequently unobserved barriers exist to implementing an SMS text messaging intervention in a way that ensures that participants receive and comprehend the messages. Our findings suggest the need for systematic monitoring and inclusion of detailed process evaluation in research studies and programmatic interventions using mobile communications to improve health.

The use of SMS text messages to provide tuberculosis-related health information is a complex intervention, as defined by having multiple, interacting components [30]. Key components of this intervention included health workers, computer servers and software, mobile telephone networks, mobile handsets, and community members; for such complex interventions, a detailed process evaluation is required to understand if all elements work together as intended [20]. We found that a significant proportion of participants never received the SMS text messages as intended, and that a large proportion of those who did receive the messages never read them. Even among those who did read the messages, a notable proportion were unable to accurately report the details of the message only 2 weeks later. Ultimately, less than a third of participants reported both receiving and retaining the tuberculosis-related information contained in the SMS text messages that were sent.

SMS text messaging interventions have been evaluated across sub-Saharan Africa for their capacity to improve medication adherence [31], support the dissemination of lab results [32], and reduce missed clinic visits [33]. However, with few exceptions [34], the existing literature does not address how often SMS text messages are received, understood, and retained by participants. In one study, which followed up on SMS text messages sent from a laboratory to 385 persons living with HIV in Uganda, only 72% of participants reported receiving the SMS text messages that were sent [34]. As in our study, participant literacy and the ability of participants to independently access SMS text messages on their phones at enrollment were associated with receiving SMS. Given these findings, future research should focus on improving accessibility to the behavioral components of the intervention through functionalities, such as automated voice calling, and, more generally, on embracing human-centered approaches to the design of mHealth interventions [35].

In our study, surprisingly, few individuals confirmed the message receipt through SMS text message reply. While a previous study in Uganda reported a 70% response rate to SMS text message containing health education quizzes [36], another large study conducted in northern Uganda involving a one-time SMS text messaging intervention found that only 23% responded, similar to what we observed in this study [37]. Although a widely cited systematic review has previously shown that 2-way SMS text messages are more effective than one-way SMS text messages in engaging patients [38], the focus of that review was on longitudinal SMS text messaging interventions for medication adherence, not on responses to a one-time communication. In a broader context that would include short-term SMS text messaging interventions to facilitate diagnostic evaluation and linkage to care, we hypothesize that SMS text message response rates—a key component of the implementation fidelity—likely also depend on other components of fidelity, including the dose, intensity, and behavioral mechanisms of the SMS text messaging intervention. Future studies should go beyond the simple process measures that we included in this study to describe these other important mediators.

The proportion of messages reported as received in this study was unexpectedly low given the widespread use of SMS text message in Uganda. Additional studies of how participants access SMS text messages are needed to understand the barriers between message initiation and participant receipt. Potential barriers at this step could include network outages and, as our qualitative data shows, malfunctioning of mobile devices and phone-sharing practices of participants. Furthermore, if mobile service providers do not provide delivery confirmations, innovative methods for assessing message receipt will be needed. In this study, simple SMS text message replies were an insufficient measure of receipt. Previous studies have shown higher levels of engagement when utilizing serial text messaging and 2-way communications [9,10,39,40] rather than one-time text messaging. Including quizzes or trivia may also improve participant response rates [36]. These strategies, along with more personalized SMS text message content [24], may increase participant engagement.

This study has a few limitations. First, we had a limited sample size that, combined with a high proportion of messages that did not reach their intended targets or convey the information intended, limited our ability to carry out stratified analyses to understand differences between subgroups. This limitation is partly moderated by our collection of qualitative responses from individuals about their experiences and interactions with the SMS text messages, which illustrate the types of barriers that participants face in engaging with mHealth interventions. Second, a programming error reduced the number of individuals who received SMS, including the subgroup for whom clinic follow-up would have been requested. This error prevented us from carrying out an analysis of the effect of different message types on participant interactions with SMS text messages and participant follow-up behaviors. In addition, it may have caused us to modestly underestimate response rates, as a previous study reported lower rates of response among those with normal results than among those with abnormal results [34]. Moreover, our programming error underscores the difficulties of ensuring successful SMS text message initiation, even with intensive quality assurance practices in place. Finally, we waited 2 weeks after triggering the initial SMS text message before attempting to contact participants to avoid interfering with parent study outcomes; this design feature may have biased the observed rate of retention of information downward, as recall error may increase with time.

This study also had several strengths. First, we had a low rate of participants lost to follow-up. We were able to interview 80% of participants by phone, and obtain, at least, some follow-up information from 90% of participants. Second, we applied innovative techniques to determine message receipt using reply SMS text messages, although the uptake of this method of message verification was extremely low. Finally, our study population included young residents of a crowded urban area with high rates of access to mobile phones [24], making our findings likely generalizable to many urban settings in sub-Saharan Africa where health-related SMS text messaging interventions are being evaluated and implemented.

Overall, this study found lower than expected levels of SMS text message receipt and retention, and substantial delays in delivery. While SMS text messages have the potential to ease communication between health workers and patients, improving the delivery cascade of SMS text messages is imperative for the success of SMS text messaging interventions. If mobile text messaging interventions are to have their full impact, innovative process measures to confirm the receipt and comprehension must be developed and applied. With better monitoring and quality improvement strategies, SMS text messaging could reach more patients more effectively, enhancing communication between patients and practitioners and building more patient-responsive health systems.

## Appendix

Multimedia Appendix 1 Tuberculosis evaluation categories and SMS message content.

Multimedia Appendix 2 Follow-up phone survey for participants who did not send an SMS reply.

Multimedia Appendix 3 Follow-up phone survey for participants who sent an SMS reply.

Multimedia Appendix 4 Predictors of sending an SMS reply message.a.

## Abbreviations

CHW: community health worker
IQR: interquartile range
mHealth: mobile health
OR: odds ratio
SMS: short message service
TB: tuberculosis
