# Supplementary material for: Text Messages Sent to Household Tuberculosis Contacts in Kampala, Uganda: Process Evaluation
Source: JMIR Mhealth Uhealth. 2018 Nov 20;6(11):e10239. doi: 10.2196/10239 (PMC6280036; doi:10.2196/10239)
Supplement: Multimedia Appendix 1 [file mhealth_v6i11e10239_app1.pdf]

Multimedia Appendix 1. Tuberculosis evaluation categories and SMS message content.

Priority [a]	Category	Indications	Message Content
1	Confirmed TB	• Any TB diagnosis based on a positive microbiological test result or clinical evaluation	(Participant name) your tests show TB. (Participant name) please come to (health center name) for TB treatment if you have not already.
2	TB clinic visit pending	• Person living with HIV, or<br>• Child under age 5, or<br>• Home sputum sample not collected when indicated, or<br>• Indeterminate lab result	(Participant name) please come to be checked for TB at (health center name) if you have not already.
3	Confirmed not TB	• 1 negative GeneXpert result, or<br>• 2 or more negative smear results	(Participant name) your tests do not show TB, but if you do not get better, reply HELP (no charges apply). Tests can miss TB if done too early.
4	No TB symptoms or risk factors	• No initial risk factors or TB symptoms known or reported by participants at the baseline	(Participant name) you do not currently have signs &/or symptoms of TB, but if you develop any signs or symptoms of TB reply HELP (no charges apply).

TB, tuberculosis; SMS, short message service.

[a]The 4 categories listed above are hierarchical, so that if a participant meets the criteria for more than one category, the message for the category highest in the table is sent. In addition, the automated message algorithm evaluates patient criteria daily, and if the patient criteria change, so that a participant is reclassified into a higher category, that message will be sent.
