# Supplementary material for: Text Messages Sent to Household Tuberculosis Contacts in Kampala, Uganda: Process Evaluation
Source: JMIR Mhealth Uhealth. 2018 Nov 20;6(11):e10239. doi: 10.2196/10239 (PMC6280036; doi:10.2196/10239)
Supplement: Multimedia Appendix 2 [file mhealth_v6i11e10239_app2.pdf]

Multimedia Appendix 3. Follow-up phone survey for participants who did not send an SMS reply.

My name is ……., I am a team member with TB contact investigation program with the Uganda National TB and Leprosy Programme and investigators from Makerere University and Yale University in the USA. A community health worker from [health center name] health center visited your home a few weeks ago to screen your household for TB symptoms. I am calling to ask a few questions as a follow-up about text messages you may have received after this visit from our study.		
1. Are you willing to answer a few questions about your text messaging experiences?	No (0)<br>Yes (1)	1.
**If participant answers No, thank participant for their time and end survey**		
2. Did you receive an SMS message about TB at some point during the last 2-3 weeks? (**Skip** to Question 7 if No)	No (0)<br>Yes (1)	2.
3. Did you read the SMS message when it first arrived?	No (0)<br>Yes (1)	3.
4. Did you read the SMS at any point? (**Skip** to Question 7 if No)	No (0)<br>Yes (1)	4.
5. Do you remember what the SMS message said? (**Skip** to Question 7 if No)	No (0)<br>Yes (1)	5.
6. What did the message say? (Ask the question open ended and then place an X next to any response(s) below that match what the participant says)		
a. Participant unable to describe message		6a.
b. Participant mentioned need to return to the clinic		6b.
c. Mention of HELP keyword		6c.
d. Mention of TB unlikely or negative status		6d.
e. Mention of TB positive status		6e.
f. Other: Please specify	6f:	
7. Please list any interesting comments from the participant or issues encountered.	7.	
