# Supplementary material for: Text Messages Sent to Household Tuberculosis Contacts in Kampala, Uganda: Process Evaluation
Source: JMIR Mhealth Uhealth. 2018 Nov 20;6(11):e10239. doi: 10.2196/10239 (PMC6280036; doi:10.2196/10239)
Supplement: Multimedia Appendix 3 [file mhealth_v6i11e10239_app3.pdf]

Multimedia Appendix 4. Follow-up phone survey for participants who sent an SMS reply.

My name is …….., I am a team member with TB contact investigation program with the Uganda National TB and Leprosy Programme and investigators from Makerere University and Yale University in the USA. A community health worker from [health center name] health center visited your home a few weeks ago to screen your household for TB symptoms. I am calling to ask a few questions as a follow-up about text messages you may have received after this visit from our study.		
1. Are you willing to answer a few questions about your text messaging experiences?	No (0)<br>Yes (1)	1.
**If participant answers No, thank participant for their time and end survey**		
2. Within the last few weeks we sent you an SMS text message about TB that you said you received. Do you remember what that message said? (**Skip** to Question 4 if No)	No (0)<br>Yes (1)	2.
3. What did the message say? (Ask the question open ended and then place an X next to any response(s) below that match what the participant says)		
a. Participant unable to describe message		3a.
b. Participant mentioned need to return to the clinic		3b.
c. Mention of HELP keyword		3c.
d. Mention of TB unlikely or negative status		3d.
e. Mention of TB positive status		3e.
f. Other: Please specify	3f:	
4. Please list any interesting comments from the participant or issues encountered.	4.	
