# Supplementary material for: Text Messages Sent to Household Tuberculosis Contacts in Kampala, Uganda: Process Evaluation
Source: JMIR Mhealth Uhealth. 2018 Nov 20;6(11):e10239. doi: 10.2196/10239 (PMC6280036; doi:10.2196/10239)
Supplement: Multimedia Appendix 4 [file mhealth_v6i11e10239_app4.pdf]

Multimedia Appendix 2. Predictors of sending an SMS reply message.[a]

	Adjusted Odds Ratio (95% CI)	*P*
Age[b]	0.59 (0.31-1.11)	.101
Personal phone ownership	13.2 (1.67-104)	.014
SMS language		
English	2.47 (0.62-9.77)	.199
Luganda	1	

[a]Adjusted for household clustering.
[b]Per 10-year increase in age.
